# Acute pancreatitis and hepatitis in an 11-year-old boy following *Mycoplasma pneumoniae* infection: case report

**DOI:** 10.3389/fped.2026.1750118

**Published:** 2026-04-02

**Authors:** Ji-Hyuk Lee

**Affiliations:** Department of Pediatrics, College of Medicine, Chungbuk National University, Cheongju, Republic of Korea

**Keywords:** child, hepatitis, *Mycoplasma pneumoniae*, pancreatitis, pneumonia

## Abstract

*Mycoplasma pneumoniae* infection is a common cause of respiratory illness in children, but extrapulmonary involvement is uncommon and may present diagnostic challenges. Among these manifestations, hepatic injury and acute pancreatitis are rarely reported in pediatric patients. We describe the case of an 11-year-old boy who developed recurrent fever and abdominal pain after initial improvement from M. pneumoniae pneumonia. On admission, he exhibited markedly elevated aspartate aminotransferase and alanine aminotransferase levels, along with significant increases in amylase and lipase. Abdominal ultrasonography demonstrated mild pancreatic swelling and thickening of the gallbladder and common bile duct walls, consistent with acute pancreatitis and associated hepatobiliary inflammation. The patient was treated with nil per os, intravenous hydration, analgesics, proton pump inhibitors, and continued azithromycin therapy. His abdominal pain gradually improved, and liver and pancreatic enzyme levels normalized over the course of hospitalization. He was discharged after 6 days, and follow-up ultrasonography confirmed complete resolution of pancreatobiliary abnormalities. This case underscores the importance of considering acute pancreatitis when abdominal pain and elevated liver enzymes develop during the course of M. pneumoniae infection, even in patients with improving respiratory symptoms. Early recognition and supportive management can help prevent complications and ensure favorable outcomes.

## Introduction

*Mycoplasma pneumoniae* is well known as a common pathogen that frequently causes interstitial pneumonia in children and adolescents (1). Beyond pulmonary disease, it may induce multisystem extrapulmonary manifestations involving skin, CNS, liver, pancreas, and hematologic systems, supported by comprehensive reviews (2). Among these, hepatic injury (hepatitis) and acute pancreatitis are uncommon but clinically significant.

Proposed mechanisms include immune-mediated injury, direct cytotoxic effects, and immune complex–mediated vascular injury (3).

Hepatic involvement associated with *M. pneumoniae* infection has been reported primarily as transient acute hepatitis characterized by elevated transaminase levels and favorable clinical outcomes. Previous pediatric studies suggest that immune-mediated hepatocellular injury, rather than direct bacterial invasion, plays a central role. Histopathologic findings in some cases have demonstrated inflammatory infiltration and hepatocellular necrosis, supporting an immunologic mechanism of liver injury (2).

In contrast, M. pneumoniae–associated acute pancreatitis remains a rare but increasingly recognized extrapulmonary manifestation. Recent case reports have described pancreatic inflammation occurring during or shortly after respiratory infection, with markedly elevated amylase and lipase levels and radiologic evidence of pancreatic swelling. The proposed pathogenesis similarly implicates immune-mediated injury, possibly riggered by circulating immune complexes or cross-reactive antibodies affecting pancreatic tissue (4, 5).

Although hepatic and pancreatic involvement have each been described individually, their simultaneous occurrence remains exceptionally uncommon, particularly in pediatric patients, underscoring the clinical significance of the present case.

In this case report, we report on the clinical course, laboratory findings, and treatment response of an 11-year-old boy who developed both acute hepatitis and acute pancreatitis following M. pneumoniae infection. This case underscores the importance of considering extrapulmonary organ involvement in M. pneumoniae infection, particularly when hepatic or pancreatic enzyme elevations are observed during respiratory illness.

## Case presentation

An otherwise healthy 11-year-old boy initially presented himself to a private clinic with fever, cough, and sputum. Chest radiography and laboratory tests led to a diagnosis of *Mycoplasma pneumoniae* pneumonia (Table 1), and azithromycin was started. The respiratory symptoms improved for 3 days, but on day 6 after diagnosis he redeveloped fever and complained of upper abdominal pain. Laboratory testing revealed elevated aspartate aminotransferase (AST)/alanine aminotransferase (ALT), and he was transferred to our hospital.

**Table 1:** Infectious work-up for differential diagnosis.

Category	Test	Method	Result
*Mycoplasma pneumoniae* (MP)	MP IgM antibody	Serology	1:320 (positive)
Respiratory viruses	Influenza A/B	PCR	Negative
	RSV	PCR	Negative
	Adenovirus	PCR	Negative
	Rhinovirus/Enterovirus	PCR	Negative
	Parainfluenza virus	PCR	Negative
	Metapneumovirus	PCR	Negative
Viral hepatitis	HAV IgM	Serology	Negative
	HBsAg	Serology	Negative
	Anti-HCV	Serology	Negative
	Ebstein-Barr virus VCA-IgM	Serology	Negative
	Cytomegalovirus-IgM	Serology	Negative

PCR, polymerase chain reaction; VCA, viral capsid antigen.

On arrival, his vital signs were stable, but he had fever of 39.0 °C and complained of nausea and epigastric pain; respiratory symptoms were not prominent. Laboratory results showed a white blood cell count of 9,880/µL (neutrophils, 13.1%), platelet count of 379 × 10³/µL, total bilirubin of 1.37 mg/dL (reference range, 0.2–1.1), AST/ALT levels of 1,657/1,301 IU/L (reference range, 5–40), amylase of 935 IU/L (reference range, 30–110), lipase of 1,581 IU/L (reference range, 13–60), lactate dehydrogenase of 3,118 IU/L (reference range, 120–300), C-reactive protein of 2.96 mg/dL (reference range, <0.03), and a Mycoplasma pneumoniae IgM titer of 1:320 and all respiratory viral panel tests were negative (Table 2). Based on these findings, acute hepatitis and acute pancreatitis due to *Mycoplasma* infection were diagnosed, and he was admitted for treatment.

**Table 2:** Day-wise clinical course and laboratory changes.

Day	Total bilirubin	AST	ALT	Amylase	Lipase	CRP	Clinical course
	(mg/dL)	(IU/L)	(IU/L)	(IU/L)	(IU/L)	(mg/dL)	
	(0.2–1.1)	(10–40)	(5–40)	(30–110)	(13–60)	(< 0.03)	
Day 1	1.37	657	1,301	935	1,581	2.96	Fever, severe epigastric pain; admission
Day 3	1.26	457	1,212	324	295	2.93	Fever subsided; abdominal pain improving
Day 6	0.58	50	398	100	96	0.63	Oral feeding resumed; clinical improvement
Day 13	0.50	29	85	106	46	0.02	Enzymes near normalization
Day 37	0.68	25	24	67	15		Complete clinical and biochemical recovery

AST, aspartate aminotransferase; ALT, alanine aminotransferase; CRP, C-reactive protein.

On hospital day 2, abdominal ultrasonography showed hepatomegaly, mild thickening of the gallbladder wall and common bile duct, and mild swelling of the pancreas. An initial workup for alternative causes of hepatitis yielded no other etiologies.

During hospitalization, azithromycin was continued for *Mycoplasma* pneumonia. For pancreatitis, he was kept nil per os and managed with analgesics, and systemic inflammation was controlled with prednisolone 1 mg/kg/day for 3 days.

From hospital day 2, the fever subsided, and respiratory symptoms remained minimal. By day 3, laboratory tests showed improvement, and abdominal pain was regressing. On day 4, oral feeding was resumed. There was no clinical deterioration, and laboratory values continued to improve. The patient was discharged on day 6. During outpatient follow-up, at 5 weeks post-admission, both abdominal ultrasonography and liver/pancreatic enzyme tests were fully normalized (Table 2).

## Discussion

*Mycoplasma pneumoniae* (MP) infection is one of the leading causes of community-acquired pneumonia in children, accounting for approximately 10%–40% of cases (1). In most cases, the disease remains confined to the respiratory system, but roughly 25% of infections may be accompanied by extrapulmonary complications affecting the liver, skin, heart, central nervous system, hematologic system, or gastrointestinal tract (2), among these, hepatitis and pancreatitis are relatively rare, and the simultaneous involvement of both organs is exceedingly uncommon.

In the existing literature, hepatic involvement in the setting of *M. pneumoniae* infection has also been reported, though estimates of the number of published hepatitis cases vary across series. MP-associated acute hepatitis has been reported in children with generally mild, transient transaminase elevations that resolve spontaneously (4, 5). Some cases with histologic evidence of hepatocellular necrosis and inflammatory cell infiltration have been documented, but the overall prognosis has usually been favorable (6, 7). On the other hand, MP-associated acute pancreatitis is far rarer. According to a previous report, approximately 30 cases of pediatric MP-related pancreatitis have been described in the literature (8). In these pediatric cases, presentation is typically fever and abdominal pain, with elevated pancreatic enzymes and imaging evidence of pancreatic abnormality.

However, Concurrent hepatic and pancreatic involvement remains exceptionally rare, especially pediatric cases in which acute hepatitis and acute pancreatitis occur simultaneously have not been described to date. In adolescents or adults, only a handful of cases exist. A 15-year-old male patient with MP pneumonia who developed both acute pancreatitis and hepatitis has been reported (9), but no further pediatric cases have appeared. In adults, Benzaquen et al. reported a case of MP infection leading to acute pancreatitis (10). Jeong et al. described an adult male who developed MP-associated hepatitis without pneumonia (11). Compared to these adult cases, the present pediatric case is one of the very rare instances in which MP infection appears to involve both the liver and pancreas concurrently.

Regarding pathophysiology, some studies classify MP-associated extrapulmonary complications into (i) direct infection type, (ii) immune-mediated (indirect) type, and (iii) vascular occlusion type (3). In the present case, the presence of serum *Mycoplasma* IgM positivity, exclusion of other viral hepatitis and autoimmune etiologies, and the rapid normalization of hepatic and pancreatic enzymes support a likely immune-mediated injury mechanism. Microvascular damage from immune complexes or autoantibodies targeting hepatocytes and pancreatic cells may have contributed to dual organ injury.

Systemic corticosteroids were administered due to concern for immune-mediated extrapulmonary involvement, given the marked elevation of transaminases and pancreatic enzymes. Although biochemical improvement began early in the disease course, the contribution of spontaneous resolution cannot be excluded. Therefore, steroid therapy should be interpreted as a supportive intervention rather than definitive evidence of treatment efficacy.

The key strength of this report is the rare concurrent presentation of hepatitis and pancreatitis following *Mycoplasma pneumoniae* infection. Although each manifestation has been reported separately, combined hepatic and pancreatic involvement remains exceptionally uncommon in children. Clinically, this case suggests that when atypical abdominal symptoms develop during or after MP infection, one should consider not only hepatic enzyme abnormalities but also pancreatitis. In patients presenting with abdominal pain, nausea, or jaundice, simultaneous measurement of liver and pancreatic enzymes and early imaging evaluation are recommended.

Limitations include the absence of contrast-enhanced CT for detailed pancreatic assessment and the lack of additional inflammatory markers such as cytokines and procalcitonin to further support immune-mediated injury. And the absence of paired IgG serology to confirm a four-fold rise represents a limitation of this case report, although the diagnosis was supported by positive IgM serology and compatible clinical features. As a single case report, broader conclusions are limited, highlighting the need for larger case series.

In summary, we describe an exceptionally rare pediatric case of simultaneous acute hepatitis and acute pancreatitis following *Mycoplasma pneumoniae* infection. This case underscores that MP infection may provoke multisystem injury via immunologic mechanisms and highlights the importance of early recognition and appropriate supportive therapy in patients with similar presentations.

## Conclusion

This case highlights that *Mycoplasma pneumoniae* infection, although primarily a respiratory illness, can rarely result in significant extrapulmonary complications, including concurrent acute hepatitis and acute pancreatitis. Clinicians should maintain a high index of suspicion for pancreatic involvement when children with MP infection develop abdominal pain or marked elevations in liver enzymes. Early assessment with liver and pancreatic enzyme measurements, along with prompt abdominal imaging when indicated, is essential for timely diagnosis and appropriate supportive management. Increased awareness of this uncommon presentation may facilitate earlier recognition and help prevent potential complications.

## Data Availability

The original contributions presented in the study are included in the article/Supplementary Material, further inquiries can be directed to the corresponding authors.

## References

[1] Narita M. Classification of extrapulmonary manifestations due to mycoplasma pneumoniae infection on the basis of possible pathogenesis. Front Microbiol. (2016) 7:23. 10.3389/FMICB.2016.00023 26858701 PMC4729911

[2] Poddighe D. Extra-pulmonary diseases related to mycoplasma pneumoniae in children: recent insights into the pathogenesis. Curr Opin Rheumatol. (2018) 30(4):380–7. 10.1097/BOR.0000000000000494 29432224

[3] Ibarra A Tanenhaus MK. The flexibility of conceptual pacts: referring expressions dynamically shift to accommodate new conceptualizations. Front Psychol. (2016) 7:561. 10.3389/fpsyg.2016.00561 27199801 PMC4843760

[4] Khan HRA Singh A Usman O Rafiq S Amin A. Acute pancreatitis: an unusual extrapulmonary manifestation of mycoplasma pneumoniae. Cureus. (2022) 14(5):e25052. 10.7759/cureus.25052 35719829 PMC9200295

[5] Valdés Lacasa T Duarte Borges MA García Marín A Gómez Cuervo C. Acute pancreatitis caused by mycoplasma pneumoniae: an unusual etiology. Clin J Gastroenterol. (2017) 10(3):279–82. 10.1007/s12328-017-0733-4 28324273

[6] Kim KW Sung JJ Tchah H Ryoo E Cho HK Sun YH Hepatitis associated with mycoplasma pneumoniae infection in Korean children: a prospective study. Korean J Pediatr. (2015) 58:211. 10.3345/kjp.2015.58.6.211 26213549 PMC4510354

[7] Song WJ Kang B Lee HP Cho J Lee HJ Choe YH. Pediatric mycoplasma pneumoniae infection presenting with acute cholestatic hepatitis and other extrapulmonary manifestations in the absence of pneumonia. Pediatr Gastroenterol Hepatol Nutr. (2017) 20:124. 10.5223/pghn.2017.20.2.124 28730137 PMC5517379

[8] Sun H Wang WQ Lin L Shao ZY Zhan L Tang LF. Case report: mycoplasma pneumoniae–associated acute pancreatitis. Front Pediatr. (2024) 12:1416189. 10.3389/fped.2024.1416189 39296668 PMC11408195

[9] Hopp E Martínez LC Díaz M Vásquez Quintero A Domínguez M Di Girolamo C Mycoplasma pneumoniae pneumonia complicated with acute pancreatitis and hepatitis: a case study. Gen. (2013) 67:106–10.

[10] Benzaquen M Lebowitz D Belenotti P Durand JM Serratrice J. Acute pancreatitis and pneumonia due to Mycoplasma pneumoniae: a case report. BMC Res Notes. (2016) 9:397. 10.1186/s13104-016-2196-y 27506562 PMC4979111

[11] Jeong HT Lee HS Lee JH Choi JH Kang YN Kim YH. Mycoplasma pneumoniae-associated hepatitis without lung involvement. Korean J Gastroenterol. (2017) 70(1):50–5. 10.4166/kjg.2017.70.1.50 28728317

